# How close are we to standardised extended *RAS* gene mutation testing? The UK NEQAS evaluation

**DOI:** 10.1136/jclinpath-2016-203822

**Published:** 2016-09-28

**Authors:** Susan D Richman, Jennifer Fairley, Rachel Butler, Zandra C Deans

**Affiliations:** 1 Department of Pathology and Tumour Biology, Leeds Institute of Cancer and Pathology, St James University Hospital, Leeds, UK; 2 UK NEQAS for Molecular Genetics, Department of Laboratory Medicine, The Royal Infirmary, Edinburgh, UK; 3 Cardiff and Vale UHB-Medical Genetics University Hospital of Wales, Cardiff, Wales, UK

**Keywords:** EQA, COLORECTAL CANCER, MOLECULAR PATHOLOGY

## Abstract

**Aims:**

Since 2008, *KRAS* mutation status in exon 2 has been used to predict response to anti-EGFR therapies. Recent evidence has demonstrated that *NRAS* status is also predictive of response. Several retrospective ‘extended *RAS*’ analyses have been performed on clinical trial material. Despite this, are we really moving towards such extended screening practice in reality?

**Methods:**

Data were analysed from four consecutive UK National External Quality Assessment Service for Molecular Genetics Colorectal cancer External Quality Assessment schemes (during the period 2014–2016), with up to 110 laboratories (worldwide) participating in each scheme. Testing of four or five tumour samples is required per scheme. Laboratories provided information on which codons were routinely screened, and provided genotyping and interpretation results for each sample.

**Results:**

At least 85% of laboratories routinely tested *KRAS* codons 12, 13 and 61. Over the four schemes, an increasing number of laboratories routinely tested *KRAS* codons 59, 117 and 146. Furthermore, more laboratories were introducing next generation sequencing technologies. The pattern of ‘extended testing’ was reassuringly similar for *NRAS*, although fewer laboratories currently test for mutations in this gene. Alarmingly, still only 36.1% and 24.1% of participating laboratories met the ACP Molecular Pathology and Diagnostics Group and American Society of Clinical Oncology guidelines, respectively, for extended *RAS* testing in the latest assessment.

**Conclusions:**

Despite recommendations in the UK and USA on extended *RAS* testing, there has clearly been, based on these results, a delay in implementation. Inadequate testing results in patients being subjected to harmful treatment regimens, which would not be the case, were routine practice altered, in line with evidence-based guidelines.

## Introduction

Over the past few years, *KRAS* mutation testing has become compulsory as a prerequisite for the treatment of patients with metastatic colorectal cancer (mCRC), with anti-EGFR monoclonal antibody (mAb) therapies such as cetuximab or panitumumab. It has been well established that patients with mutations in exon 2 (codons 12 and 13) of *KRAS* fail to respond to anti-EGFR therapy and display inferior outcomes when therapy is combined with oxaliplatin-based chemotherapy.^1–5^ It is also apparent that a significant proportion of exon 2 wild-type (WT) patients gain no benefit from anti-EGFR therapy. This has led to the need for additional refinement of the patient population receiving such therapy, in order to better select patients for treatment, and equally identify those who will gain no benefit from a toxic drug regimen.

Recently available data, resulting primarily from the reanalysis of several large randomised controlled trials (RCTs), have provided evidence for the introduction of an extended *RAS* testing panel, to include *NRAS* testing and also to cover significantly more mutation hotspots, in *KRAS*. A prospective–retrospective analysis was carried out on the phase III PRIME study^6^ to evaluate the treatment effect of panitumumab-FOLFOX4 (Pan-FOLFOX4), when compared with FOLFOX4 alone, in patients with *RAS*-WT (*KRAS* and *NRAS* exons 2, 3 and 4 WT) and patients with *RAS-*WT plus *BRAF*-WT (*KRAS* and *NRAS* exons 2, 3 and 4 and *BRAF* exon 15 WT). Both overall survival (OS) and progression-free survival (PFS) were significantly increased with Pan-FOLFOX4. Furthermore, 17% of the 639 patients with mCRC, with no *KRAS* mutation in exon 2, had additional *RAS* mutations, which were associated with worse PFS and OS, when treated with Pan-FOLFOX4.

Last year, data were published from the phase III CRYSTAL trial^7^ where extended *RAS* testing was carried out on the mCRC *KRAS* exon 2 WT trial population. As was seen in the PRIME study, both OS and PFS were significantly increased in the patients receiving cetuximab plus FOLFIRI, compared with those receiving FOLFIRI alone. The percentage of patients who carried *RAS* mutations in addition to *KRAS* exon 2 was 14.7%, which is similar to the level identified in the patients in the PRIME trial (17%).

The data from these two large trials, in combination with the data from a further 13 articles,^8–19^ formed the basis of the recent American Society of Clinical Oncology (ASCO) Provisional Clinical Opinion (PCO) update.^20^ The recommendation of the PCO was that *KRAS* exon 2 (codons 12 and 13) mutations should be assessed in addition to *KRAS* exon 3 (codons 59 and 61) and exon 4 (codons 117 and 146), along with *NRAS* exon 2 (codons 12 and 13), exon 3 (codons 59 and 61) and exon 4 (codons 117 and 146).

Current European guidelines for prescribing anti-EGFR monoclonal antibody therapy (http://www.ema.europa.eu/docs/en_GB/document_library/Summary_of_opinion/human/000741/WC500144827.pdf) state that ‘Vectibix (Panitumumab) is indicated for the treatment of adult patients with wild-type *RAS* metastatic colorectal cancer (mCRC)’, without specifically stating which codons must be tested. In the UK, however, the Association of Clinical Pathologists Molecular Pathology and Diagnostics Group devised a set of guidelines, directed at UK practice and more specifically within the National Health Service (NHS).^21^ The recommendation was that *RAS* testing for treatment with anti-EGFR therapies should include, as a minimum, *KRAS* codons 12, 13, 59, 61, 117 and 146 and *NRAS* codons 12, 13, 59 and 61. As these codons account for over 99% of *RAS-*activating mutations,^22^ UK National External Quality Assessment Service (UK NEQAS) for Molecular Genetics has provided assessment of molecular testing of colorectal cancer since 2008^23^ and has determined that it is good practice for all of these to be covered by laboratories participating in their External Quality Assessment (EQA) schemes.

Given that there is now adequate data available confirming the need to perform extended *RAS* testing, we report here on the very varied range of *RAS* (*KRAS* and *NRAS*) codons being routinely tested for mutation detection, across four UK NEQAS for Molecular Genetics Colorectal cancer EQA schemes, with laboratory testing being performed between October 2014 and November 2015, in up to 110 UK and international laboratories.

## Materials and methods

The UK NEQAS for Molecular Genetics EQA scheme for the molecular genetic analysis of colorectal cancer is provided twice during each 12-month period, with approximately 6 months between each run. Multiple formalin-fixed, paraffin-embedded (FFPE) colorectal tumour tissue blocks were sourced to include a number of WT and *RAS* mutations, and variable sample processing/fixation processes into the scheme. To ensure the samples distributed for the EQA scheme gave reportable accurate *RAS* results, each sample was tested in two independent, validating laboratories using two different testing methods, prior to being sent out to participating laboratories. Consent was in place to allow the use of this excess pathological material in the UK NEQAS EQA scheme. Data were available from the two runs delivered in 2014–2015 (Runs 1 and 2) and the two runs delivered during 2015–2016 (Runs 1 and 2). Each year, participating laboratories are sent samples with five fictitious clinical scenarios in Run 1 and four in Run 2. Laboratories are expected to test these nine samples in accordance with local laboratory practice and report results for both *KRAS* and *NRAS* testing. It was deemed optional as to whether laboratories chose to report *BRAF* and *PIK3CA* testing results. Each laboratory was required to stipulate the preferred sample type: rolled sections for each case; rolled sections plus a slide-mounted section or only slide-mounted sections, and all requests were met by UK NEQAS. An estimate of tumour percentage was expected from each laboratory, for each tumour sample, or a statement indicating that tumour assessment was not carried out. None of the tumour samples distributed had less than 20% tumour cell content. Each laboratory was also required to provide a clinical interpretation of their results according to the clinical cases provided, and state the methodologies used for mutation detection.

## Results

### Scheme participation

Eighty-six laboratories registered to participate in Run 1 of the 2014–2015 scheme, and of these, 84 submitted results. Ninety-one laboratories registered to participate in Run 2, and again 84 submitted results. In the 2015–2016 scheme, 101 laboratories registered to participate in Run 1, and 99 submitted results, and 108/120 registered laboratories returned results in Run 2. The two laboratories in Run 1 and six laboratories in Run 2 failing to submit reports in the 2014–2015 scheme followed the correct protocol, and contacted the Scheme to advise them of the non-submission of results. Likewise, the two laboratories in Run 1 and seven laboratories in Run 2 failing to report results for the 2015–2016 scheme also followed the correct protocol. However, one laboratory in Run 2 of the 2014–2015 scheme and five laboratories in Run 2 of the 2015–2016 scheme were deemed poor performing laboratories due to non-submission in accordance with the published guidelines.

### Sample type tested

The distribution of sample types requested did not vary much across the four schemes. Table 1 shows the percentage of laboratories requesting mounted FFPE tumour sections only, rolled sections of tissue plus a slide-mounted section or rolled sections only.

**Table 1:** Distribution of sample types requested by the laboratories, registering for each of the four schemes

	Run 1 2014–15 (n=86)	Run 2 2014–15 (n=91)	Run 1 2015–16 (n=101)	Run 2 2015–16 (n=120)
Mounted FFPE section only	34 (40%)	34 (37%)	37 (37%)	46 (38%)
Rolled section plus mounted FFPE section	36 (42%)	40 (44%)	46 (46%)	53 (44%)
Rolled section only	16 (19%)	17 (19%)	18 (18%)	21 (18%)

For each run, not every laboratory requesting material went on to complete their submission

FFPE, formalin-fixed, paraffin-embedded.

### Regions of *KRAS* and *NRAS* tested

A comparison of the regions of *KRAS* and *NRAS* covered by mutation screening across the three schemes was performed. For *KRAS*, there was an increase in the number of laboratories testing each codon, with the largest increases seen in codons 59, 117 and 146. There was also an increase in the number of laboratories stating just the exons tested (exons 2, 3 and 4), indicative of an increase in the number of laboratories moving to a next generation sequencing (NGS) platform (table 2). For *NRAS*, a very similar pattern was seen across all codons; however, clearly, there are still fewer laboratories testing *NRAS*, although a smaller proportion of participating laboratories is failing to report results for *NRAS* (table 3).

**Table 2:** Percentage of laboratories in each scheme run, stating the codons (or exons) that were covered in their screening panels for *KRAS*

Codons or exons tested	*KRAS* Run 1 (2014–15) n=84	*KRAS* Run 2 (2014–15) n=84	*KRAS* Run 1 (2015–16) n=99	*KRAS* Run 2 (2015–16) n=108
Codons 12/13	82.1	89.3	81.8	76.9
Codon 59	14.3	15.5	20.2	29.6
Codon 61	73.8	85.5	71.7	69.4
Codon 117	16.7	41.6	47.5	50.9
Codon 146	35.7	52.4	54.5	54.6
Exon 2	9.5	9.5	12.1	15.7
Exon 3	9.5	9.5	12.1	15.7
Exon 4	4.8	4.8	10.1	13.9
Not specified	8.3	1.2	6.1	7.4

**Table 3:** Percentage of laboratories in each scheme run, stating the codons (or exons) that were covered in their screening panels for *NRAS*

Codons or exons tested	*NRAS* Run 1 (2014–15) n=84	*NRAS* Run 2 (2014–15) n=84	*NRAS* Run 1 (2015–16) n=99	*NRAS* Run 2 (2015–16) n=108
Codons 12/13	58.3	73.8	68.7	66.7
Codon 59	14.3	22.6	23.2	28.7
Codon 61	71.4	73.8	65.7	63.9
Codon 117	15.5	20.2	21.2	21.3
Codon 146	21.4	26.2	30.3	29.6
Exon 2	10.7	10.7	13.1	15.7
Exon 3	10.7	10.7	13.1	15.7
Exon 4	6	16.7	10.1	11.1
Not tested	22.6	13.1	12.1	10.2
Not specified	8.3	1.2	6.1	7.4

We investigated the gene regions tested in the most recent scheme (Runs 1 and 2 of 2015–2016), which was provided following the updated UK guidelines.^21^ Each laboratory was expected to state which regions of *KRAS* and *NRAS* were covered by their screening protocol. Despite this requirement, five laboratories in Run 1 and eight laboratories in Run 2 failed to provide this information on their diagnostic clinical report.

There was a slight decrease in the percentage of laboratories not testing for mutations in *NRAS*, dropping from 12/99 (12.1%) to 11/108 (10.2%) from Run 1 to Run 2. In Run 1, there were two samples harbouring *NRAS* mutations: one containing a c.35G>T and one containing a c.181C>A mutation. In Run 2, there was one sample harbouring a c.34 G>T mutation. Thus, 12 and 11 laboratories failed to report these mutations in Runs 1 and 2, respectively.

Tables 4 and 5 show the proportion of laboratories meeting, or failing to meet, the UK and US guidelines for extended RAS testing respectively. Despite the increase seen across the two runs, there was still a significant proportion of laboratories that was effectively undertesting.

**Table 4:** The percentages of laboratories either meeting the UK guidelines for extended *RAS* testing, or indeed overtesting or undertesting

	Run 1 (n=99)	Run 2 (n=108)
Percentage of laboratories exactly following UK guidelines	6.1	25.9
Percentage of laboratories overtesting	19.2	10.2
Percentage of laboratories undertesting	68.7	55.6
Percentage of laboratories where the testing panel was undetermined	6	8.3
TOTAL percentage of laboratories testing at least in accordance with UK guidelines	25.3	36.1

Adding together the laboratories following the UK guidelines and those overtesting gives the total percentage of laboratories meeting or exceeding the minimum requirements. The UK guidelines suggest the following coverage: *KRAS* codons 12, 13, 59, 61, 117 and 146 in addition to *NRAS* codons 12, 13, 59 and 61.

**Table 5:** The percentages of laboratories either meeting the US guidelines for extended *RAS* testing, or indeed overtesting or undertesting

	Run 1 (n=99)	Run 2 (n=108)
Percentage of laboratories exactly following ASCO guidelines	4	13
Percentage of laboratories overtesting	15.2	11.1
Percentage of laboratories undertesting	74.8	67.6
Percentage of laboratories where the testing panel was undetermined	6	8.3
TOTAL percentage of laboratories testing at least in accordance with ASCO guidelines	19.6	24.1

Adding together the laboratories following the US guidelines and those overtesting gives the total percentage of laboratories meeting or exceeding the minimum requirements. The current American Society of Clinical Oncology (ASCO) guidelines state that the coverage should include *KRAS* exon 2 (codons 12 and 13), *KRAS* exon 3 (codons 59 and 61), *KRAS* exon 4 (codons 117 and 146), in addition to *NRAS* exon 2 (codons 12 and 13), *NRAS* exon 3 (codons 59 and 61) and *NRAS* exon 4 (codons 117 and 146).

Taking into account the current UK NEQAS for Molecular Genetics recommendations, only 24/99 (24.2%) and 39/108 (36.1%) of laboratories are meeting these minimum requirements in Runs 1 and 2, respectively.

When the current ASCO guidelines are taken into account, only 19/99 (19.2%) of laboratories met the recommendations in Run 1, with this rising to 26/108 (24.1%) in Run 2.

## Discussion

In an era of personalised medicine, it is becoming increasingly important to identify which patients will respond to specific drugs, and equally, which will gain no benefit. Furthermore, additional stratification is required to ensure the greatest benefits are obtained, once patients are selected for individual treatment. It was established in 2008 that *KRAS* mutation was a negative predictive biomarker of response to panitumumab^3^ and cetuximab.^1^ ^24^ In these studies, only *KRAS* codons 12 and 13 were tested for the presence of pathogenic mutations. The PICCOLO trial, run across the UK, was one of the first mCRC randomised trials to introduce prospective mutation testing, allowing randomisation based on mutation status.^25^ Again, only *KRAS* status, at codons 12, 13 and 61, was assessed prospectively. Based on the evidence of the low response rate to anti-EGFR therapies, the group also carried out a retrospective analysis of additional mutation hotspots (*BRAF* codon 600; *NRAS* codons 12, 13 and 61; *KRAS* codon 146; *PIK3CA* codons 542, 545-6 and 1047). Patients who were WT at all loci (‘all wild-type’) demonstrated a high response rate to panitumumab (70/160 (44%)) and also an improved PFS (HR 0.68; 95% CI 0.53 to 0.86), whereas patients whose tumours contained any mutation demonstrated a detrimental effect from panitumumab, in terms of PFS (HR 1.20; 95% CI 0.83 to 1.74). During 2015, there was a publication of the extended *RAS* testing retrospectively carried out on patients in the OPUS trial.^26^ Only 26% of the 118 evaluable patients were found to harbour an additional *RAS* mutation. Patients with WT tumours demonstrated an improved objective response with the addition of cetuximab to FOLFOX4 (58% vs 29%; OR 3.3, 95% CI 1.36 to 8.7; p=0.0084), whereas those with any mutation derived no benefit, and indeed demonstrated a detrimental effect. These data, in combination with the extended *RAS* testing in CRYSTAL and PRIME, have highlighted the need for extended mutation testing, particularly where there is the potential of a detrimental effect on the patient.

Laboratories proving a clinical service in terms of *RAS* mutation screening should be participating in a regular EQA scheme, to ensure adequate quality measures are met. The UK NEQAS for Molecular Genetics Colorectal cancer EQA scheme has observed an increase in the number of laboratories participating and in the breadth of codons screened by participants over the course of the past four EQA runs (October 2014 to November 2015). It was noted that not all laboratories carry out a pathology review of each tumour sample. Almost 20% of participating laboratories requested rolled sections only for testing, and were thus unable to assess the tumour content, as requested in the scheme. These laboratories were not penalised, providing an indication was given on the laboratory report, that this was the case. UK NEQAS would rather assess each laboratory's routine process; so, if they do not routinely assess tumour content, yet report clinical results, it is preferable that this is assessed by the EQA provider, rather than routine processing be amended for scheme participation. Most markedly have been increases in the number of laboratories now including *KRAS* codons 59, 117 and 146 in their screening panels. Furthermore, there has been a substantial increase in the number of laboratories including *NRAS* testing into the testing strategies. Increased implementation of NGS panel testing was seen across the four runs examined. In Run 1 of 2014–2015, only 9.5% of laboratories were using NGS to screen for mutations on *KRAS*, and 11.9% were using NGS to screen *NRAS*. This increased to 23.1% in Run 2 of 2015–2016, for both *KRAS* and *NRAS*. It was observed that the format of reporting NGS results was highly variable, and many reports did not state the gene panel or sequencing platform used. As this is not a scheme requirement, laboratories were not penalised for failing to provide this information. The implementation of gene panel testing is clearly becoming more routine practice, as testing requirements increase. This will become more challenging as the demand for large-panel gene mutation screening on small, diagnostic biopsies, where the limiting factor will be the tissue sample itself, increases.

As previously mentioned, in both USA and UK, there are now published guidelines on extended *RAS* testing.^20^ ^21^ Alarmingly, we have shown here that of the laboratories (n=108) participating in the most recent EQA scheme, only 24.1% of laboratories met the ASCO guidelines and 36.1% met the UK guidelines. Figures based on the CRYSTAL and PRIME^6^ ^7^ studies would suggest that although 40% of patients with mCRC are likely to have a *KRAS* exon 2 mutation, there are still between 14.7% and 17% of patients with an additional *RAS* mutation, providing strong evidence for extended *RAS* testing. In the USA, there are currently two FDA-approved mutation screening kits (Therascreen RGD PCR kit and the Roche cobas *KRAS* mutation testing kit), both of which only cover seen mutations in codons 12 and 13 of *KRAS*. These kits are clearly a very long way from meeting the ASCO guidelines, yet may have to be used in certain laboratories. In Europe, the European Medicines Agency (EMA) is not prescriptive as to the testing methodologies or scope of testing, which enables laboratories to introduce in-house developed tests or other commercially available kits, which incorporate wider *RAS* testing. Each laboratory therefore has the choice of determining the extent of testing in accordance with best-practice guidance. However, the implementation of new tests requires validation and verification, which are costly, and many testing centres have no resources for such validation. This may go some way to explaining the limited and different testing strategies employed across the scheme participants. Clearly, there is still a long way to go before these guidelines are followed in routine practice, and until this happens, we will see a larger than necessary patient cohort, subjected to the detrimental effects of their anti-EGFR therapies, which would not have been prescribed, had an extended *RAS* panel been incorporated into practice.

**Take home messages**

- We have provided evidence of the nature of current *RAS* testing, across 100 global laboratories, in the context of an international quality assurance scheme.
- We have demonstrated the alarming lack of full extended *RAS* testing, in accordance to both UK and US guidelines.
- Practice must be altered to bring laboratories in line with these evidence-based guidelines to ultimately provide a high standard of patient care.
